# A Novel 1000 MPa Grade Ultrafine-Grained Dual-Phase Press Hardening Steel with Superior Oxidation Resistance and High Ductility

**DOI:** 10.3390/ma16175994

**Published:** 2023-08-31

**Authors:** Dapeng Yang, Jiawei Liang, Junlong Zhou, Xin Xu, Zhiping Hu, Xingli Gu, Guodong Wang

**Affiliations:** 1 State Key Laboratory of Rolling and Automation, Northeastern University, Shenyang 110819, China; yangdapeng@ral.neu.edu.cn (D.Y.); zhou_junlong0208@126.com (J.Z.); wanggd@mail.neu.edu.cn (G.W.); 2 Technology Center of Angang Steel Company Limited, 63 Wuyi Road, Anshan 114009, China; huzhiping900401@126.com (Z.H.); gu_xingli@126.com (X.G.)

**Keywords:** press hardening steel, dual phase, oxidation resistance, ductility, bendability

## Abstract

1000 MPa grade low-carbon martensite press hardening steels (PHS) are widely used in energy-absorbing domains of automotive parts, such as the bottom of a B-pillar. To prevent oxide scale formation during hot forming, this PHS is often required to be protected by an additional Al–Si coating. In addition, although the low carbon martensitic microstructure grants it excellent bending toughness, the ductility tends to be limited. In this study, a novel 1000 MPa grade ultrafine-grained (UFG) martensite–ferrite (F–M) dual-phase (DP) PHS with superior oxidation resistance was designed using tailored additions of Cr, Mn, and Si, and refining the initial microstructure. Only 0.55 ± 0.18 μm thick oxide film is formed in the designed steel during austenitizing heating and stamping, which is significantly lower than the 24.6 ± 3.1 μm thick oxide film formed in conventional 1000 MPa grade low-carbon martensite PHS under the identical condition. The superior oxidation resistance of designed steel can be attributed to the rapid formation of the protective Si-rich, Cr-rich, and Mn-rich oxide layers during annealing. Moreover, due to the presence of ferrite and ultrafine microstructure, the designed steel also shows a significant improvement in ductility from 8.5% to 16.8% without sacrificing strength and bending toughness compared with conventional 1000 MPa grade low-carbon martensite PHS.

## 1. Introduction

PHS is widely used for automobile structural component due to its impressive mechanical properties and no springback after forming [1,2]. Based on the application, PHS is mainly divided into anti-intrusion PHS and high energy-absorbing PHS [3,4,5]. The former is usually required to possess high yield strength and ultimate tensile strength. The most typical example is the 22MnB5 with a yield strength and ultimate tensile strength of over 1100 MPa and 1500 MPa, respectively. However, its bending toughness tends to be limited [3,4]. The latter usually exhibits impressive bending toughness, which endows it with extraordinary energy-absorption ability during collision [5,6]. A typical example is 1000 MPa grade low-carbon martensitic PHS [5]. In actual automotive components, these two PHSs are usually required to be combined using laser welding followed by integral hot stamping, to obtain gradient performance parts, which can realize the combination of high strength-toughness [7]. Such integrated hot stamping of anti-intrusive PHS with high energy-absorbing PHS is widely used in tailor hot-stamped, laser-welded door rings [8].

Typically, the manufacturing process for PHSs involves annealing the cold-rolled sheet above the austenitizing temperature for a few minutes followed by die-quenching [3,4]. However, the surface of the plate is easily oxidized during the annealing process, which deteriorates the surface quality and brings decarburization [9]. Moreover, the oxide scales that fall off during the hot forming also accelerate stamping tool wearing [10]. Therefore, conventional PHSs, such as 22MnB5, usually require a protective Al–Si coating to prevent the oxide scale formation during the hot forming. However, this approach imposes additional costs and deteriorates the bending toughness [11,12]. To overcome this drawback, optimizing the chemical composition of PHS to improve oxidation resistance is an effective strategy. For example, a novel 1800 MPa grade Cr-alloyed medium-Mn PHS developed by Li et al. exhibited an oxidization layer with a thickness of less than 3 μm after hot forming at 750~810 °C for 5 min; such a tremendously reduced oxidation result is believed to be caused by the lower soaking temperature employed for the hot forming and the formation of dense Cr/Al/Si oxide band at the bottom of the oxidation layer [13]. Ding et al. achieved a 1700 MPa grade F–M dual phase PHS with high oxidation resistance by adding Cr and Si, in which the oxide film thickness was less than 1.6 μm after hot forming at 825 °C for 3 min [14]. It was believed that the enhanced oxidation resistance is attributed to the lower hot forming temperature and the denser/thicker Si/Cr-rich oxide band formed at the bottom of layer. Moreover, this hot stamping also exhibits good ductility of ~11.2% due to the introduction of the ferrite phase. Unfortunately, almost all studies on high-temperature oxidation resistance have focused on 1500~2000 MPa grade anti-intrusion PHSs, while related studies on energy-absorbing PHSs are extremely lacking, especially 1000 MPa high energy-absorbing PHSs, which are usually matched with 1500~2000 MPa anti-intrusion PHSs for integrated hot stamping to form gradient performance parts, such as tailor hot-stamped laser-welded door rings [7,8]. Therefore, to improve the overall antioxidant performance of the part, the oxidation resistance of 1000 MPa high energy-absorption PHS also needs to be emphasized. It is worth mentioning that, in addition to low oxidation resistance, the microstructure of conventional 1000 MPa high energy-absorbing PHS is usually dominated by low-carbon martensite, which exhibits excellent toughness; the ductility, however, tends to be limited, about 6% [8].

In fact, the essence of the antioxidant properties achieved by the addition of Si, Cr, and Mn in PHSs is that the higher affinity between these elements and O than that between Fe and O means these elements preferentially combine with oxygen to form Si-rich, Cr-rich, and Mn-rich oxides [15,16]. Since these oxides usually exhibit a denser structure and are firmly bonded with the substrate, the diffusion of Fe and O atoms is effectively suppressed, thus eliminating the formation of the loose Fe-oxide layer [13,15,17]. Moreover, these elements interact with each other during the oxidation process. For example, Si is beneficial to reduce the critical Cr content for forming continuous Cr_2_O_3_ film and, thus, contributes to the formation of continuous Cr_2_O_3_ film [18]. Therefore, in order to improve the oxidation resistance, these elements can be added simultaneously to the alloy [8]. In addition, the oxidation resistance of the alloy is also related to the initial structure. Refinement of the initial structure can further promote the rapid formation of the Si-rich, Cr-rich, and Mn-rich oxide layers due to the presence of numerous grain boundaries and phase boundaries, which can produce a short-circuit diffusion path and, thus, contribute to the diffusion of Si, Cr, and Mn atoms [19,20]. Moreover, the fine initial structure also can offer a high density of preferred nucleation sites for austenite formation and, thus, refine the final microstructure, which contributes to improving the deformation damage resistance and enhancing the mechanical properties of the alloy [21,22]. 

Based on the above discussion, to overcome the low oxidation resistance and poor ductility of the conventional 1000 MPa low-carbon martensite high energy-absorbing HPS, our study tried to optimize the alloy compositions (mainly by adding a certain amount of Si, Cr, and Mn elements) combined with a fine initial structure to obtain a 1000 MPa grade UFG F–M dual-phase HPS to achieve a combination of high oxidation resistance and high ductility. Moreover, the oxidation and mechanical behaviors of experimental steels were further investigated.

## 2. Materials Design and Experimental Procedures

**Figure 1:**
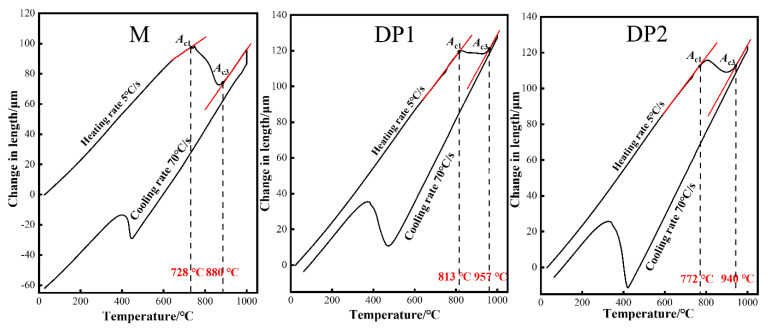
Dilatometric curve of three experimental steels after the thermal cycle.

The chemical composition of designed PHSs (referred as DP1 and DP2) and comparison 1000 MPa grade low-carbon martensite PHS (referred as M steel) are exhibited in Table 1. The main characteristic of the chemical composition of DP1 and DP2 is the addition of 1.6 wt.% Si and 1.5 wt.% Cr, which promotes the protective Cr-rich and Si-rich oxide formation during the annealing process and, therefore, prevents the oxidation of the substrate [12,13]. Moreover, Si is also beneficial to reduce the critical Cr content for forming continuous Cr_2_O_3_ film and, thus, contributes to the formation of continuous Cr_2_O_3_ film [18]. Compared to DP1, the higher Mn content in DP2 contributes to more Mn-rich oxide formation during the annealing process, thus further improving the oxidation resistance [16]. Additionally, the addition of higher Mn content is beneficial to suppressing the ferrite phase transformation during the air-cooling process after hot-rolling, thus facilitating the achievement of a finer hot/cold rolled microstructure [23]. A finer initial structure not only facilitates the rapid formation of the Si-rich, Cr-rich, and Mn-rich oxide layer during the annealing process [18,19], but it also contributes to the refinement of the final microstructure. The latter is beneficial for mechanical properties [21,22]. It is worth mentioning that both DP1 and DP2 exhibit high *A*_c1_ and *A*_c3_ temperatures due to the addition of 1.6 wt.% Si according to the dilatometric curves shown in Figure 1 [24], which allows the achievement of a ferrite-austenite dual-phase structure at 900 °C for 5 min. Furthermore, DP2 exhibits lower *A*_c1_ and *A*_c3_ temperatures compared to DP1 due to the higher content of Mn added to DP2, which can reduce *A*_c1_ and *A*_c3_ temperatures [13]. 

**Figure 2:**
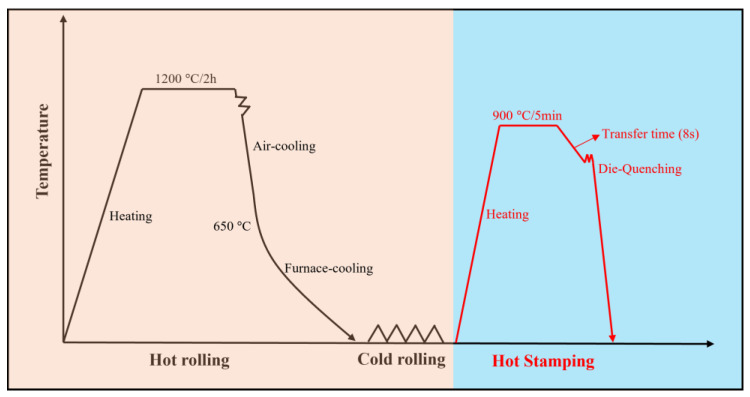
Graphical view of experimental steels preparation process.

The designed steels were manufactured as an ingot using vacuum induction melting and then forged into a slab with the dimension of 500 × 140 × 35 mm^3^. The slabs with the dimension of 150 × 140 × 35 mm^3^ were heated to 1200 °C for 2 h and hot-rolled to a dimension of 1750 × 140 × 3 mm^3^ after 7 passes. Subsequently, the hot-rolled steel plates were air-cooled to 650 °C and then cooled from 650 °C to room temperature at a box furnace to simulate coiling in the industry. Finally, the hot-rolled plates with a thickness of 3 mm were pickled to remove the oxide layer formed during hot-rolling and then cold-rolled to 1 mm in thickness. In the case of heat treatment, the designed steels with the dimension of 230 × 140 × 1 mm^3^ were annealed at 900 °C for 5 min in a furnace without protective atmosphere, and then transferred to the press for die-quenching. The corresponding graphical view of experimental steels preparation process is shown in Figure 2.

The actual *A*_c1_ and *A*_c3_ temperature was measured using a Bahr DIL805A/D dilatometry, in which a sample with the dimension of 10 × 4 × 1 mm^3^ was heated at 5 °C/s to 900 °C, held for 5 min, and then cooled at 70 °C/s. The microstructure of samples with the RD (rolling direction)–ND (normal direction) plane was characterized using a ZEISS ULTRA 55 field emission scanning electron microscope (SEM). Prior to this, specimen surfaces (lengths of 15 and 1 mm in RD and ND directions, respectively) were mechanically polished and subsequently etched in 4% nital solution for 10 s. Regarding the oxidation resistance evaluation, the surface and cross-sections of the oxide film were analyzed using SEM incorporated with the Xplore dispersive spectrum (EDS). Besides, phase identification studies of oxide film were carried out on samples with RD–TD (transverse direction) plane with dimensions of 15 × 15 mm^2^ using X’pert PRO X-ray diffraction (XRD) apparatus with a Cu target (λ = 0.15406 nm) at ambient temperature in the 2θ range of 15~90° and step size of 2°/min. To further identify the phase of the oxide film, the ESCALAB 250Xi X-ray photoelectron spectroscopy (XPS) is used for analysis of the chemical compositions of the oxide film, in which the RD and TD dimensions of the samples are 15 and 15 mm, respectively.

Tensile tests were conducted at a constant crosshead speed of 1 mm/min on dog-bone-shaped specimens with a width of 6.25 mm and a gauge length of 25 mm using a CMT 5105 pc-controlled mechanical testing system (USA, MTS Co., Ltd., Gwangyang-si, Republic of Korea) at room temperature. The average value of the tensile test results was calculated from three samples for each case. Bending tests were conducted on specimens with the dimension of 60 × 60 × 1 mm^3^ in accordance with VDA 238-100 standard. The punch is bent vertically to the rolling direction of the sample.

## 3. Results 

### 3.1. Initial Microstructure of the Experimental Steels

**Figure 3:**
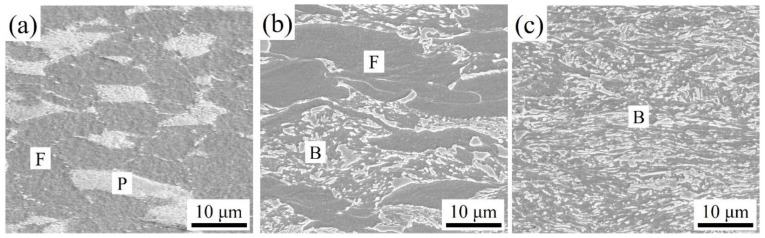
Typical morphologies after cold-rolling: (**a**) M; (**b**) DP1; (**c**) DP2.

The microstructure of three experimental steels after cold rolling is shown in Figure 3. The microstructure of M steel mainly consists of deformed ferrite (referred as F) and pearlite (referred as P). DP1 is dominated by deformed ferrite and bainite (referred as B). Compared to DP1, the microstructure of DP2 is characterized as fully deformed bainite due to the addition of higher Mn content, which suppresses the ferrite phase transformation during the air-cooling process after hot-rolling [17].

### 3.2. Morphology of the Oxide Film of the Experimental Steels after Press Hardening

**Figure 4:**
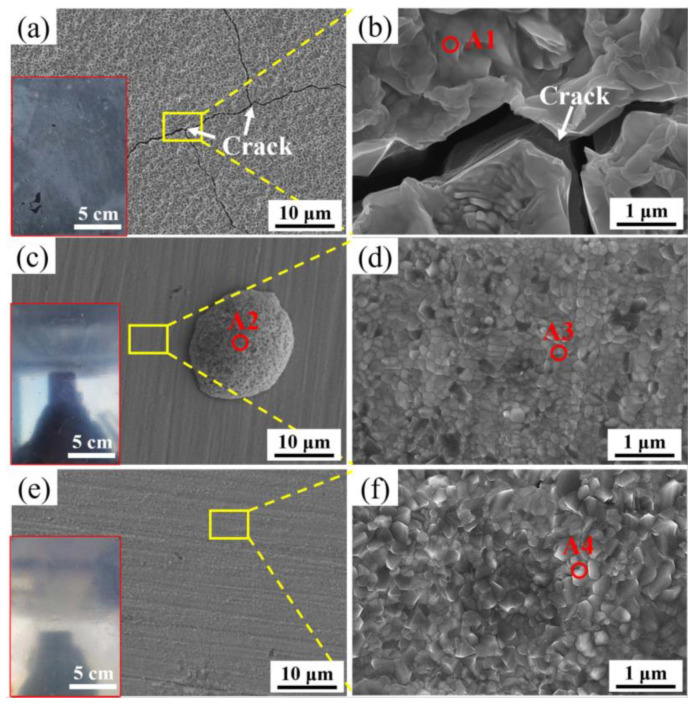
Typical morphologies of the oxide film of (**a**,**b**) M, (**c**,**d**) DP1, and (**e**,**f**) DP2 after press hardening.

Figure 4 shows the morphology of the oxide film surface of three experimental steels after press hardening. For M steel, the significant oxidation behavior leads to the loss of metallic luster of the sample surface (Figure 4a). The magnification of the oxide film reveals that there are obvious cracks in the oxide film (Figure 4b). The chemical composition of the A1 region is analyzed by EDS and reveals that this oxide is rich in O and Fe with an atomic fraction ratio close to 1.5, as shown in Table 2. This indicates that the outermost layer of the oxide film is Fe_2_O_3_. By contrast, the sample surface of the DP1 steel still shows a metallic luster (Figure 4c), which indicates that the oxidation behavior is significantly suppressed. It is noteworthy that the oxide film of DP1 exists in two regions, one of which is characterized by large-sized porous oxide nodules (A2 region) with an area proportion of 3.2%, while the remaining dominant region (area proportion of 96.8%) is distributed with dense oxide particles with an average size of 120 ± 15 nm (A3 region), as shown in Figure 4c,d. EDS analysis revealed only enrichment of O and Fe in the A2 region and the corresponding atomic fraction ratio between O and Fe is close to 1.5, which indicates the outermost layer of the large-sized porous oxides region is Fe_2_O_3_. The A3 region is enriched with Fe, O, Cr, and Mn elements. The macroscopic morphology of DP2 also exhibits a distinct metallic luster after press hardening (Figure 4e), which also indicates a significant improvement in oxidation resistance. Further amplification of the oxide film revealed that the oxide film is dominated by dense oxide particles with an average size of 185 ± 31 nm (Figure 4f). This oxide (A4 region) is also identified by EDS to be obviously enriched in Fe, O, Cr, and Mn elements. Moreover, the enrichment of Mn in the A4 region is more obvious compared to that in the A3 region, indicating that more Mn-rich oxides exist in the oxide film of DP2 compared with DP1. 

**Figure 5:**
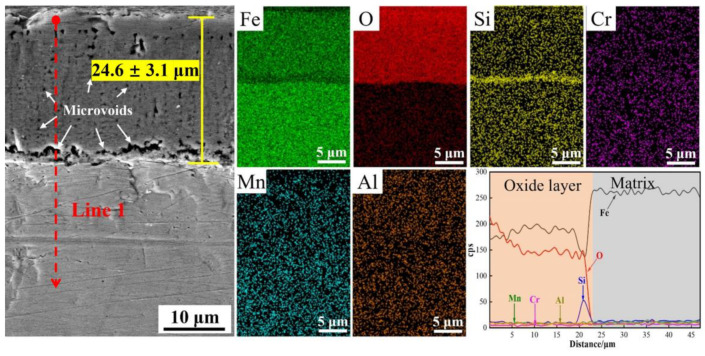
The cross-sectional morphology and corresponding critical elemental distribution of oxide film for M after press hardening.

The morphology of the cross-section of the oxide film and the corresponding critical elemental distribution of the oxide film for M steel are exhibited in Figure 5. M steel shows a thicker oxide film with an average thickness of 24.6 ± 3.1 μm. Moreover, a large number of microvoids (shown by white arrows) can be observed within the oxide film and at the interface between the oxide film and the matrix, indicating that the oxide films formed in M steel are relatively loose. According to the distribution of critical elements in the cross-sectional and line 1, only the Si-rich oxide layer forms at the interface between the outer Fe-rich oxide layer and the matrix.

**Figure 6:**
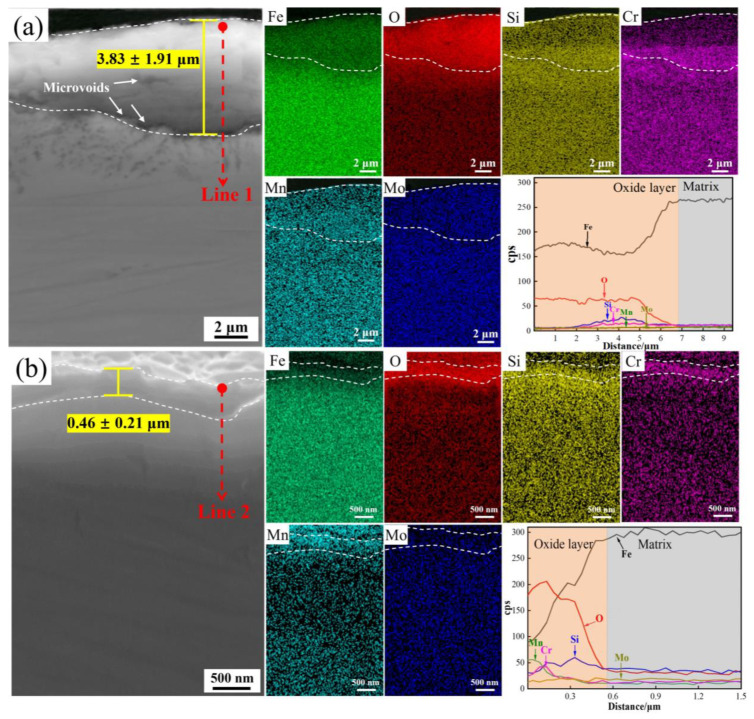
The cross-sectional morphology and corresponding critical elemental distribution of oxide film in DP1 after press hardening. (**a**) porous Fe-oxide nodules; (**b**) oxide particles.

According to Figure 4c,d, two morphologies of oxides are present in DP1 after press hardening: a low area proportion of porous Fe-oxide nodules and a dominant area proportion of oxide particles. The cross-sectional morphology and corresponding critical elemental distribution of the former are shown in Figure 6a. The average thickness of Fe-oxide nodules is 3.83 ± 1.91 µm, and a large number of microvoids can be observed within the oxide film and at the interface between the oxide film and the matrix, indicating the incompact oxide film. The distribution of the critical elements in the cross-section of the oxide film and line 1 shows that the bottom of the oxide film is enriched in Fe, Si, Cr, and O, while the outside of the oxide film only exhibits an enrichment of Fe and O. By contrast, the cross-sectional morphology and corresponding critical elemental distribution of the latter (oxide particles) are shown in Figure 6b. The oxide film exhibits an extremely thin oxide film with a thickness of only 0.46 ± 0.21 μm. Moreover, no defects were found within the oxide film, which indicates that the oxide film is extremely compact. According to the distribution of critical elements on the cross-section and line 2, it is clear that the oxide film is significantly enriched in Si, Cr, and Mn, which indicates Si-rich, Cr-rich, and Mn-rich oxides exist in the oxide film. Moreover, Si seems to be more enriched on the bottom of the oxide film, while Cr tends to be enriched in the middle, and Mn is enriched on the outside. In summary, although porous Fe-oxide nodules are still present in the oxide film of DP1, considering that thin oxide particles still dominate the oxide film of DP1, it can be concluded that the oxidation resistance of DP1 is significantly improved compared to that of M.

**Figure 7:**
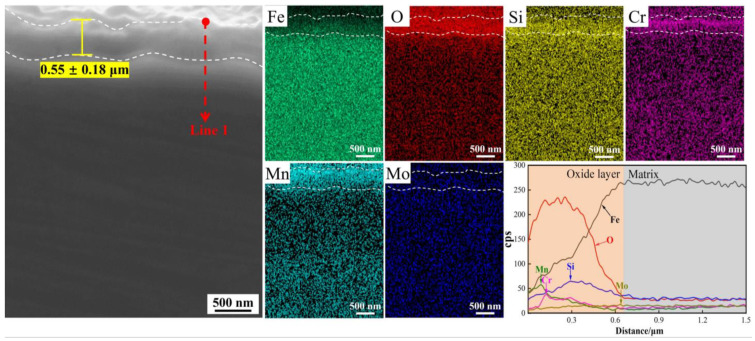
The cross-sectional morphology and corresponding critical elemental distribution of oxide film in DP2 after press hardening.

The cross-sectional morphology and corresponding critical elemental distribution of oxide film for DP2 after press hardening are shown in Figure 7. DP2 also exhibits an extremely compact oxide film with an average thickness of 0.55 ± 0.18 μm. Moreover, Si also seems to be more enriched on the bottom of the oxide film, while Cr tends to be enriched in the middle and Mn is enriched on the outside.

### 3.3. Phase Composition of the Oxide Film of the Experimental Steels after Press Hardening

**Figure 8:**
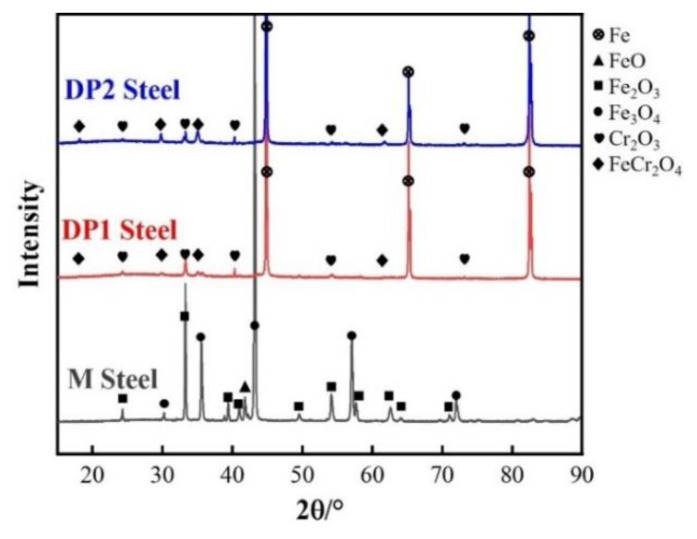
XRD diffraction patterns of M, DP1, and DP2 steels after press hardening.

To clarify the phase composition of the oxide film of M, DP1, and DP2, the XRD analysis was conducted, as shown in Figure 8. The oxide film of M steel is mainly composed of FeO, Fe_2_O_3,_ and Fe_3_O_4_. For conventional low-alloy steels, the outer layer of the Fe-oxide film is Fe_2_O_3_, the middle layer is Fe_3_O_4_, and the innermost layer is FeO [25]. However, the diffraction peak of the Si-rich oxide is not presented, which might be attributed to the low content of this oxide. For DP1 and DP2 steels, the diffraction peaks are strongly influenced by the matrix because the oxide film is too thin, thus showing strong diffraction peaks of Fe. Moreover, slight FeCr_2_O_4_ and Cr_2_O_3_ diffraction peaks can still be observed. However, considering the low content of Mn-rich and Si-rich oxides and the limitations of the equipment measurement accuracy, Mn-rich and Si-rich oxides are not detected.

**Figure 9:**
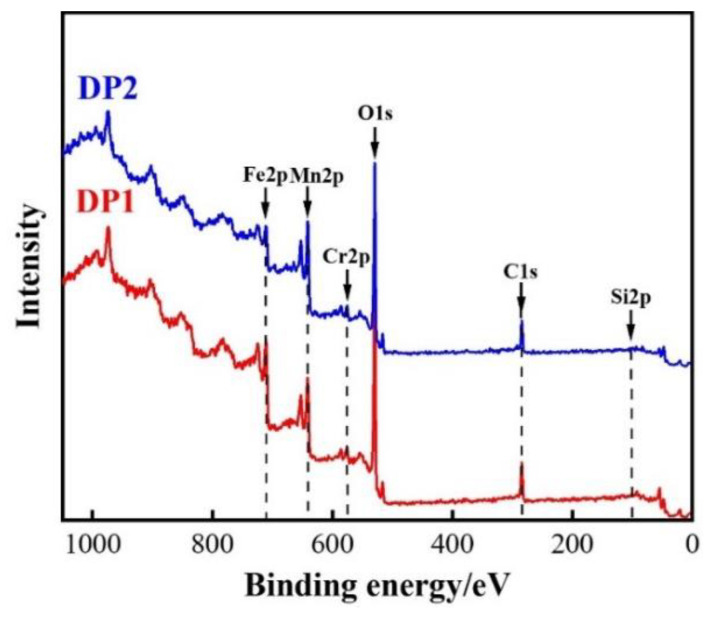
XPS diffraction patterns of the oxide film in DP1 and DP2 steels after press hardening.

To better assess the chemical compositions of the oxide film surface, the complete XPS spectra of DP1 and DP2 after press hardening are exhibited in Figure 9. The results revealed that the oxide film surface of DP1 and DP2 mainly contained Fe, C, O, Cr, and Mn. The C element detected in XPS is related to the adsorption of CO_2_ by the oxide film, which is inevitable [26]. Interestingly, the Si element is not detected, which implies that Si-rich oxide is mainly enriched within the oxide film. The related mass fraction of crucial elements, i.e., Fe, O, Cr, and Mn, in the oxide film surface are summarized in Table 3. The oxide film surface of both DP1 and DP2 exhibits obviously higher Mn content than Cr content (Table 3), indicating that the Mn-rich oxide tends to be more towards the outside of the oxide film compared to Cr-rich oxides. These results correspond to the elemental distribution results shown in Figure 6 and Figure 7. In addition to this, it is noteworthy that the DP2 oxide film surface presents higher Mn content and lower Fe content compared with DP1. This result further suggests that more Mn-rich oxides are formed on the oxide film surface of DP2 compared with that of DP1, and confirms that DP2 exhibits better oxidation resistance than DP1, which effectively inhibits the outward diffusion of Fe ions.

**Figure 10:**
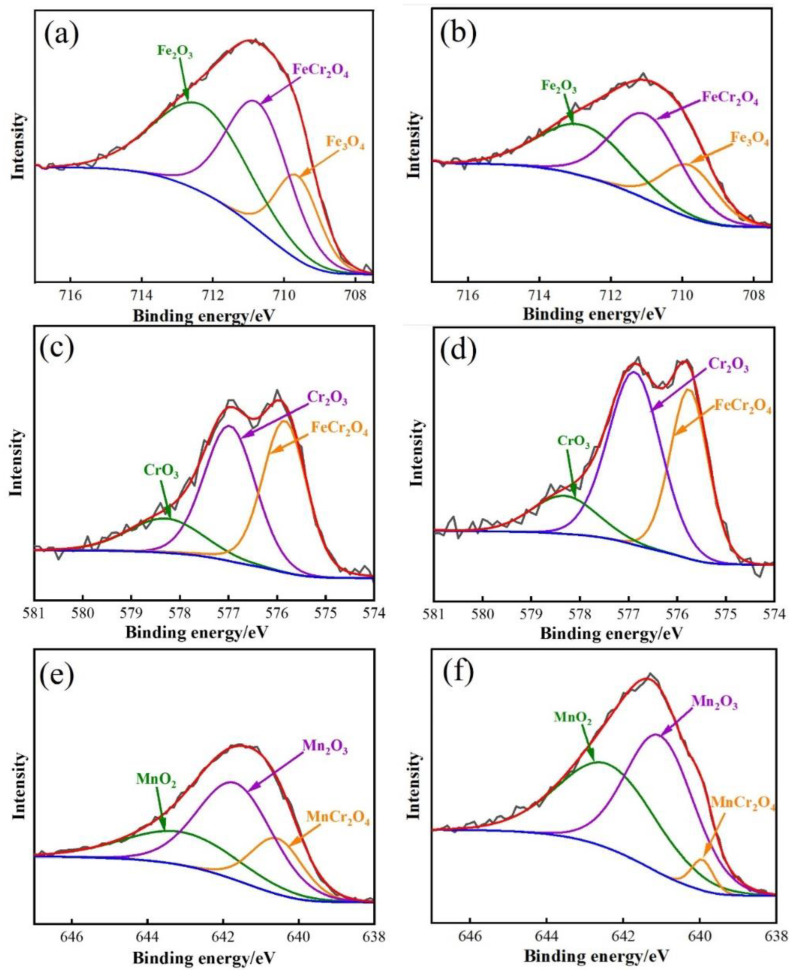
High-resolution XPS spectra of the oxide film in DP1 and DP2 steels after press hardening: (**a**,**c**,**e**) Fe 2p3/2, Cr 2p3/2, and Mn 2p3/2 of DP1; (**b**,**d**,**f**) Fe 2p3/2, Cr 2p3/2, and Mn 2p3/2 of DP2.

To further analyze the oxidation products of each element, the high-resolution XPS spectra of Fe 2p3/2, Cr 2p3/2, and Mn 2p3/2 of DP1 and DP2 steel are shown in Figure 10. The high-resolution Fe 2p3/2 core level spectra shown reveal the presence of three components with binding energies of 712.0 ± 0.3 eV, 710.9 ± 0.4 eV, and 709.6 ± 0.5 eV, respectively (Figure 10a,b), which correspond to the Fe_2_O_3_, FeCr_2_O_4_, and Fe_3_O_4_ oxides, respectively [17,18]. Regarding the high-resolution Cr 2p3/2 core level spectrum (Figure 10c,d), it also can be fitted with three components with binding energies of 578.7 ± 0.5 eV, 576.7 ± 0.3 eV, and 576 ± 0.5 eV, respectively, which correspond to CrO_3_, Cr_2_O_3_ and FeCr_2_O_4_ oxides, respectively [17,18]. Similarly, the binding energies of the three components shown in the high-resolution Mn 2p3/2 core level spectrum are 643.0 ± 0.2 eV, 641.5 ± 0.2 eV, and 640.4 ± 0.2 eV, respectively (Figure 10e,f), which correspond to the MnO_2_, Mn_2_O_3_, and MnCr_2_O_4_ oxides, respectively [17,18]. In summary, Fe-rich, Cr-rich, and Mn-rich oxides with multiple chemical states are present on the oxide film surface of DP1 and DP2 after press hardening.

### 3.4. Microstructural and Mechanical Properties of the Experimental Steels after Press Hardening

**Figure 11:**
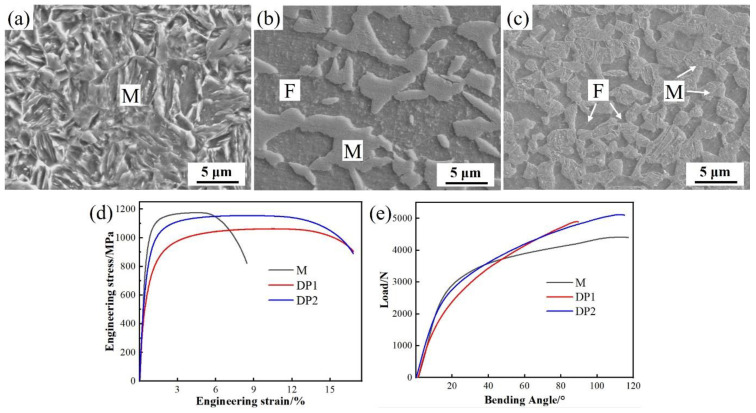
Typical SEM morphologies of M (**a**), DP1 (**b**), and DP2 (**c**), as well as corresponding engineering stress–strain curve (**d**) and bending load–angle curve (**e**) after press hardening.

From the above results, the designed steels exhibit excellent oxidation resistance; however, their mechanical properties cannot be ignored. Figure 11 shows the SEM morphologies, engineering stress–strain curve, and bending load–angle curve of the experimental steels after press hardening. The microstructure of M is dominated by martensite (Figure 11a). Regarding DP1 (Figure 11b), it is mainly characterized by ferrite and martensite with a content of 52% and 48%, respectively. DP2 also shows ferrite and martensite with a content of 35% and 65%, respectively (Figure 11c). Moreover, compared to DP1, DP2 exhibits a UFG microstructure with average grain sizes of 1.4 and 2.1 μm for ferrite and martensite, respectively. Regarding mechanical properties (Figure 11d,e), DP2 exhibits the most superior comprehensive mechanical properties among the three steels. Compared to M steel, DP2 steel exhibits comparable ultimate tensile strength and bending toughness; the ductility is significantly enhanced by 93%, from 8.6% to 16.5%. Moreover, DP2 also shows obvious advantages in ultimate tensile strength and bending toughness compared to DP1.

## 4. Discussion

### 4.1. Enhanced Oxidation Resistance Mechanism in DP2 Steel

For short-time high-temperature oxidation behavior, the rapid formation of a dense oxide film on the plate surface is the key to obtaining excellent high-temperature oxidation resistance, which can effectively prevent the outward diffusion of Fe ions and the inward diffusion of O ions, thus preventing the matrix against oxidation. In general, the oxides formed by other elements, such as Si, Cr, and Mn, are usually more densely structured than Fe-oxides and, therefore, provide better high-temperature oxidation resistance [13,15,27]. According to thermodynamic theory, the affinity between O and other elements in an alloy follows the order of Si > Mn > Cr > Fe at the temperature of 900 °C [15]. In other words, O is more likely to combine with these elements to form Si-rich, Mn-rich, and Cr-rich oxide at the early stage of oxidation. 

**Figure 12:**
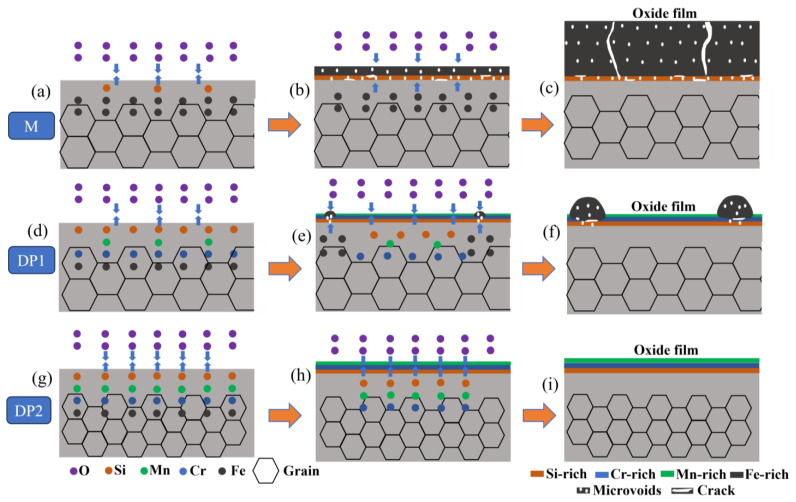
Schematic diagram of the oxidation behavior of M, DP1, and DP2 during the annealing process. (**a**–**c**): The porous Si-rich oxide layer formed by M steel with low Si content cannot prevent the diffusion of Fe and Oxygen atoms. (**d**–**f**): The coarse grains and small amounts of Mn addition in DP1 result in a lack of protection in localized areas of the oxide film, which leads to the formation of Fe-oxide nodules. (**g**–**i**): The fine grains combined with a certain amount of Si/Cr/Mn addition in DP2 contribute to the rapid formation of the protective Si-rich, Cr-rich and Mn-rich oxide layers, which significantly inhibits the Fe-oxide layer formation.

To clearly illustrate the oxidative behavior during annealing, the schematic diagrams of the oxidative behavior of M, DP1, and DP2 steels during annealing are shown in Figure 12. For M steel, the small amount of Si addition cannot form a dense Si-rich oxide layer, which corresponds to the results of the porous Si-rich oxide layer formation, as shown in Figure 5. This porous Si-rich oxide layer cannot effectively prevent the diffusion of Fe and O ions, thus promoting the Fe-oxide film formation on the outside of the Si-rich oxide layer. Moreover, this formed Fe-oxide layer also contains a large number of microvoids and cracks (Figure 4 and Figure 5), which also cannot prevent the subsequent oxidation behavior of the substrate. As a result, an extremely thick oxide film of 24.6 ± 3.1 μm is formed on the surface of the M steel after press hardening. 

For DP1 and DP2 steels with a certain content of Si, Cr, and Mn elements, according to the thermodynamic theory mentioned above, O first combines with Si to form Si-rich oxides, then with Mn to form Mn-rich oxide, and finally with Cr to form Cr-rich oxide. However, Mn-rich oxides usually tend to be more located outside of the oxide film compared to Cr-rich oxides because the diffusion coefficient of Mn through the oxide film is much higher than that of Cr in iron-based alloys [16]. This explains the oxide layer structure of DP1 and DP2; i.e., Mn-rich oxides, Cr-rich oxides, and Si-rich oxides are located at the outer, middle and bottom of the oxide film, respectively (Figure 6 and Figure 7). The oxidation resistance of alloys is related to the content of chemical components. Compared to M steels, DP1 and DP2 steel with higher content of Si and Cr forms the Si-rich oxide and Cr-rich oxide layer that is generally more protective [8]. Continuous and dense Si-rich oxide and Cr-rich oxide layer of DP1 and DP2 are demonstrated in Figure 6 and Figure 7. Additionally, Cr is considered to promote the formation of Mn-rich oxides, especially in Fe–Cr alloys where Mn-oxide formation is extremely pronounced [16,28]. This phenomenon is also confirmed in our study (Figure 6 and Figure 7; Table 2 and Table 3); i.e., a significant enrichment of Mn can be observed in the outermost portion of the oxide layer. In conclusion, the preferential formation of continuous and dense Si-rich, Cr-rich, and Mn-rich oxide layers in DP1 and DP2 steels significantly suppressed the outward diffusion of Fe ions and the inward diffusion of O ions, which is the key factor for extremely thin oxide film formation in DP1 and DP2 after press hardening.

However, DP1 and DP2 still exhibit some differences in the oxide film. For DP1, although most regions of the oxide film in DP1 are dominated by dense oxide particles, large-sized porous Fe-oxides nodules are still present in local regions (Figure 4c). This phenomenon can be attributed to the lack of sufficient protection of the oxide film in local regions, resulting in the diffusion of Fe and O ions and, thereby, the formation of porous Fe-oxides [29]. Due to the fast growth rate of porous Fe-oxides, large-size Fe-oxides nodules are eventually developed in local regions of DP1 [30]. Considering that such large-sized porous Fe-oxide nodules are probably peeling off during hot forming due to the unstable interface between it and the substrate, thus deteriorating the surface quality of the steel plate. Therefore, it is necessary to avoid these large-sized porous iron oxide nodules formation. By contrast, the oxide film of DP2 exhibits completely dense oxide particles, which indicates the better high-temperature oxidation resistance of DP2 compared to DP1 (Figure 4e). Moreover, this better high-temperature oxidation resistance of DP2 compared to DP1 can also be reflected by the lower Fe content on the oxide film surface shown in Table 3. The enhanced high-temperature oxidation resistance compared to DP1 can be attributed to the differences in chemical composition and initial microstructure between them. Compared to DP1, DP2 has a higher content of Mn, which undoubtedly promotes more Mn-rich oxide formation, thus further enhancing the high-temperature oxidation resistance of DP2 [16]. In this study, the more Mn-rich oxide formation of DP2 can be confirmed by EDS and XPS results (Table 2 and Table 3), i.e., higher Mn content in the oxide film of DP2 than DP1. In addition to the chemical composition, the high-temperature oxidation resistance is also related to the initial microstructure. For steels with a certain amount of Si, Cr, and Mn added, the finer initial structure contains more grain boundaries and consequently produces a short-circuit diffusion path that is beneficial to the rapid formation of a dense protective oxide film [18,19]. Compared to the ferrite–bainite microstructure of DP1, the complete bainite microstructure of DP2 is generally considered to contain more phase boundaries (similar to finer grain sizes), which facilitates diffusion of Si, Cr, and Mn elements and, thus, promotes the rapid formation of Si-rich, Cr-rich, and Mn-rich oxide particles. The combined effect of these two factors leads to a more rapid formation of the compact oxide film with more Mn-rich oxides in DP2 compared to DP1 during the initial stage of oxidation, which contributes to further improving the high-temperature oxidation resistance of DP2 and, thus, suppresses the formation of large-sized Fe-oxides nodules.

### 4.2. Superior Mechanical Properties of the DP2 Steel

**Figure 13:**
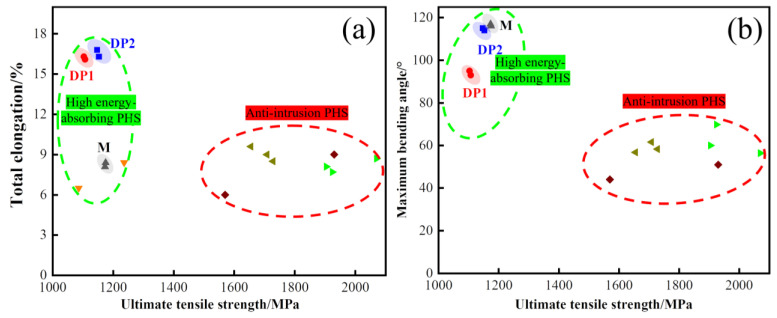
Comparison of the mechanical properties of the experimental steels with high energy-absorbing PHSs and anti-intrusion PHSs reported in the literature [4,8,31,32]: (**a**) Total elongation against ultimate tensile strength; (**b**) Maximum bending angle against ultimate tensile strength. The green dotted and red dotted circles are the mechanical properties of high energy-absorbing PHSs and anti-intrusion PHSs, respectively.

**Figure 14:**
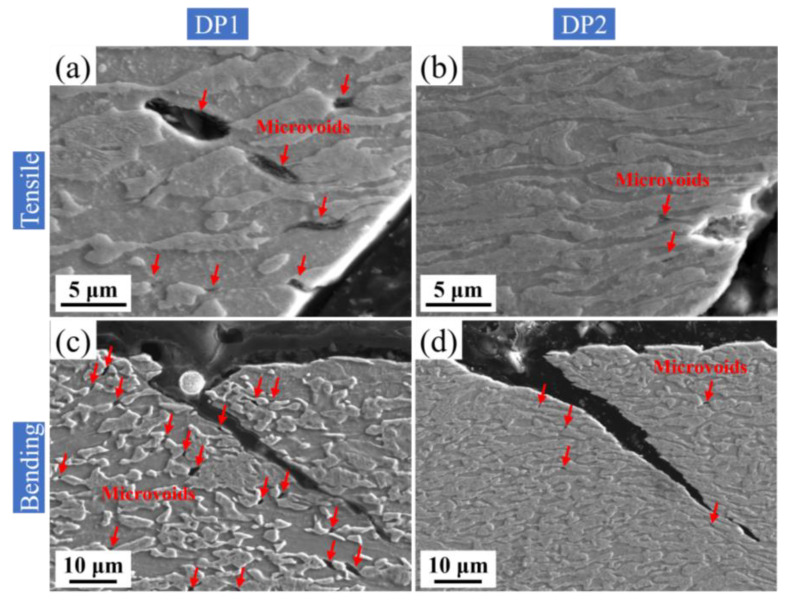
SEM images of DP1 and DP2 after tensile (**a**,**b**) and bending failure (**c**,**d**). Microvoids are noted by red arrows.

DP2 not only shows excellent high-temperature oxidation resistance, but also superior comprehensive mechanical properties. Figure 13 shows the comparison of the mechanical properties of the experimental steels with high energy-absorbing PHSs and anti-intrusion PHSs reported in the literature [4,8,31,32]. It is worth mentioning that although the thickness of the present experimental steel~1 mm is less than that of the comparison PHSs~1.5 mm, which is favorable to the maximum bending angle increasing (usually less than 10°) [33]. This means that the DP2 still shows excellent advantages in ductility-bending toughness, which contributes to a further enhancement of energy absorption during collision [8]. In particular, DP2 exhibits a significant 93% improvement in ductility compared to M without sacrificing strength and bending toughness. The enhanced ductility of DP2 compared to M can be attributed to the presence of ferrite in DP2. It is widely accepted that ferrite has higher dislocation multiplication capacity than martensite, which promotes a more durable strain hardening during deformation, thus contributing to ductility improvement [34,35]. Furthermore, DP2 exhibits fine microstructure after press hardening. Such fine microstructure of DP2 can be attributed to its fine-scale initial microstructure, i.e., cold-rolled bainite, which can offer high density of preferred nucleation sites for austenite formation, and, thus, enhances the efficiency of pinning the migration of ferrite grain boundaries during intercritical annealing [20,21]. Achieving grain refinement by tailoring a fine initial microstructure is widely reported in DP steels [20,21]. Moreover, refining the microstructure of DP steels increases strength and promotes a uniform distribution of microstrain during deformation, which helps to suppress strain localization near the martensite boundaries and contributes to inhibiting ferrite-martensite interface decohesion formation [36,37]. Moreover, grain refinement also facilitates martensite deformability and inhibits the premature formation of martensite cracking [38]. To demonstrate this, the SEM images of DP1 and DP2 after tensile and bending failure were revealed, as shown in Figure 14. It is clearly observed that a large number of microvoids with large sizes (1~5 μm) caused by ferrite/martensite interfacial decohesion can be observed close to tensile (Figure 14a) and bending fracture (Figure 14c) in DP1. By contrast, only a few microvoids with small sizes (less than 1 μm) caused by ferrite/martensite interfacial decohesion can be observed near the tensile (Figure 14b) and bending fracture (Figure 14d) in DP2. It is worth noting that the bending failure morphology comparison between DP1 and DP2 is still based on the higher bending angle experienced in DP2 (Figure 14c,d). Moreover, almost no martensite cracks can be observed near the tensile (Figure 14b) and bending fracture (Figure 14d) in DP2, although the martensite is significantly elongated, which indicates the high deformability of martensite. Therefore, the ultrafine microstructure of DP2 significantly suppresses the ferrite/martensite interfacial decohesion and martensite crack initiation during the deformation, which contributes to ductility and bending toughness enhancement. In summary, the fine ferrite-martensite dual-phase microstructure is the key to the excellent comprehensive mechanical properties of DP2.

## 5. Conclusions

In this study, to overcome the low oxidation resistance and poor ductility of the conventional 1000 MPa low-carbon martensite HPS, two novel 1000 MPa grade dual-phase PHSs (DP1 and DP2) with excellent oxidation resistance with high ductility were developed. The oxidation resistance and mechanical properties difference between DP1, DP2, and M steel were systematically investigated. The main results are as follows:

(1) DP2 steel exhibits the best oxidation resistance among the three steels. It shows only 0.55 ± 0.18 μm thick oxide film after annealing at 900 °C for 5 min, which is much lower than that formed in M steel ~24.6 ± 3.1 μm under the identical condition.
(2) The excellent oxidation resistance of DP2 can be attributed to the addition of a certain amount of Si, Cr, and Mn elements combined with the fine cold-rolled bainite initial microstructure, which contributes to the rapid formation of the protective Si-rich, Cr-rich, and Mn-rich oxide layers, thus significantly suppressing the Fe-oxides formation.
(3) DP2 exhibits a significant 93% improvement in ductility, from 8.6% to 16.5%, compared to M without sacrificing strength and bending toughness. The superior comprehensive mechanical properties of DP2 can be attributed to the ultrafine ferrite-martensite dual-phase microstructure.
(4) DP2 exhibits excellent oxidation resistance and excellent mechanical properties. However, during the actual hot-forming process, a certain amount of strain will be created in localized areas of the steel plate. However, the effect of this high-temperature predeformation on the microstructure, mechanical properties and, antioxidant properties of F–M dual-phase press hardening steel is still unknown, which will be investigated in future efforts.

## Figures and Tables

**Table 1:** Chemical compositions (wt.%) of three experimental steels.

Steels	C	Si	Mn	V	Cr	Mo	Al	Ti	B
M	0.08	0.4	1.6		0.07		0.05	0.03	0.03
DP1	0.135	1.6	0.7	0.15	1.5	0.2			
DP2	0.09	1.6	2.0	0.15	1.5	0.2			

**Table 2:** The chemical composition of the marked regions.

Marked Regions	Mass Fraction/wt.%				
	Fe	O	Si	Cr	Mn
A1	64.2	28.1	0.1	/	0.2
A2	62.5	29.5	0.1	0.1	0.2
A3	68.6	11.0	1.3	4.7	5.6
A4	59.9	13.9	1.4	4.6	13.1

**Table 3:** The chemical composition of oxide film surface for DP1 and DP2 by XPS analysis.

Steels	Mass Fraction/wt.%				
	Fe	O	Si	Cr	Mn
DP1	13.3	52.4	/	2.9	11.6
DP2	8.3	50.1	/	3.3	19.4
